# Automatic detection of head and neck squamous cell carcinoma on histologic slides using hyperspectral microscopic imaging

**DOI:** 10.1117/1.JBO.27.4.046501

**Published:** 2022-04-29

**Authors:** Ling Ma, James V. Little, Amy Y. Chen, Larry Myers, Baran D. Sumer, Baowei Fei

**Affiliations:** a University of Texas at Dallas, Department of Bioengineering, Richardson, Texas, United States; b Tianjin University, State Key Laboratory of Precision Measurement Technology and Instruments, Tianjin, China; c Emory University School of Medicine, Department of Pathology and Laboratory Medicine, Atlanta, Georgia, United States; d Emory University School of Medicine, Department of Otolaryngology, Atlanta, Georgia, United States; e The University of Texas Southwestern Medical Center, Department of Otolaryngology, Dallas, Texas, United States; f The University of Texas Southwestern Medical Center, Advanced Imaging Research Center, Dallas, Texas, United States; g The University of Texas Southwestern Medical Center, Department of Radiology, Dallas, Texas, United States

**Keywords:** hyperspectral imaging, squamous cell carcinoma, nuclei segmentation, support vector machine, convolutional neural network, classification

## Abstract

**Significance:**

Automatic, fast, and accurate identification of cancer on histologic slides has many applications in oncologic pathology.

**Aim:**

The purpose of this study is to investigate hyperspectral imaging (HSI) for automatic detection of head and neck cancer nuclei in histologic slides, as well as cancer region identification based on nuclei detection.

**Approach:**

A customized hyperspectral microscopic imaging system was developed and used to scan histologic slides from 20 patients with squamous cell carcinoma (SCC). Hyperspectral images and red, green, and blue (RGB) images of the histologic slides with the same field of view were obtained and registered. A principal component analysis-based nuclei segmentation method was developed to extract nuclei patches from the hyperspectral images and the coregistered RGB images. Spectra-based support vector machine and patch-based convolutional neural networks (CNNs) were implemented for nuclei classification. The CNNs were trained with RGB patches (RGB-CNN) and hyperspectral patches (HSI-CNN) of the segmented nuclei and the utility of the extra spectral information provided by HSI was evaluated. Furthermore, cancer region identification was implemented by image-wise classification based on the percentage of cancerous nuclei detected in each image.

**Results:**

RGB-CNN, which mainly used the spatial information of nuclei, resulted in a 0.81 validation accuracy and 0.74 testing accuracy. HSI-CNN, which utilized the spatial and spectral features of the nuclei, showed significant improvement in classification performance and achieved 0.89 validation accuracy as well as 0.82 testing accuracy. Furthermore, the image-wise cancer region identification based on nuclei detection could generally improve the cancer detection rate.

**Conclusions:**

We demonstrated that the morphological and spectral information contribute to SCC nuclei differentiation and that the spectral information within hyperspectral images could improve classification performance.

## 1 Introduction

**Fig. 1:**
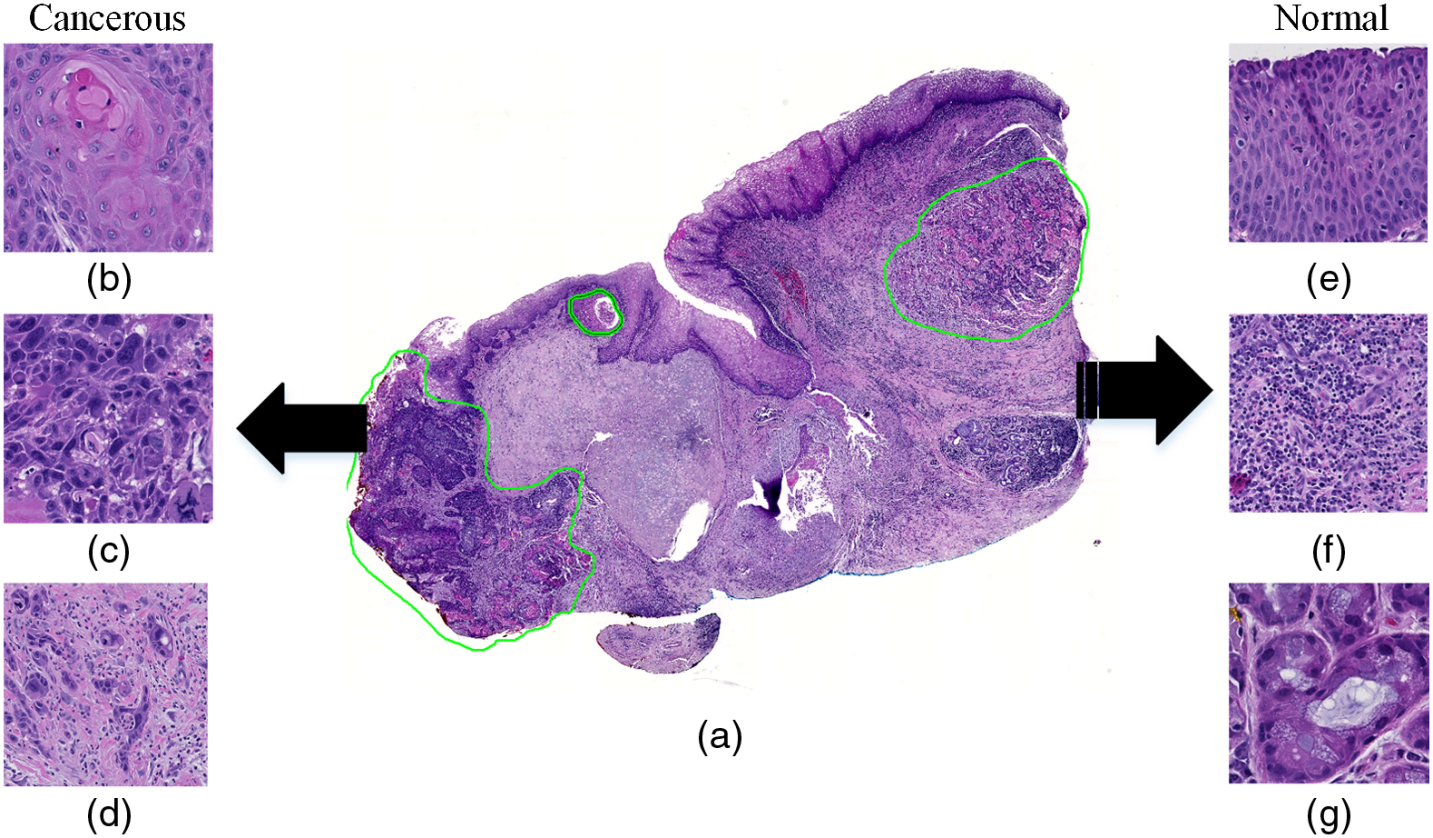
Anatomical diversity of cancerous and normal tissue in one histologic slide. (a) A digital histologic image of an hematoxylin and eosin (H&E)-stained slide of SCC at the larynx. The green contour indicates cancer, and the rest of the tissue is normal. The patches of cancerous and normal tissue shown on the left and right panels are extracted from this image (a). (b)–(d) Patches showing various histological features of SCC, including keratinizing SCC, enlarged SCC nuclei with shape variation, and SCC with chronic inflammation (from top to bottom). (e)–(g) Patches showing different normal structures, including stratified squamous epithelium, tissue with chronic inflammation, and salivary glands (from top to bottom).

Head and neck cancer (HNC) is the sixth most common cancer worldwide. Squamous cell carcinoma (SCC), which is a major type of cancer at the original sites of the upper aerodigestive tract, takes about 90% of HNC cases.^1^^–^^3^ It can occur in multiple organs including the nasopharynx, oral cavity, oropharynx, nasal cavity, paranasal sinuses, hypopharynx, larynx, and trachea.^4^^–^^6^ And histologic slides of SCC tissues are important for its histopathological analysis and diagnosis. Computer-aided pathology has been emerging in recent years. It is intended to provide a fast, reproducible, and quantitative diagnosis. Many studies have been carried out to investigate automatic cancer detection in digitalized histologic images using deep learning approaches.^7^^–^^10^ In these studies, image patches cropped from the whole-slide images were used to train a deep and rather complex neural network architecture, which learned to recognize the sophisticated histological structures all over the slides, as shown in Fig. 1. Due to the anatomical diversity of the histologic slides, these works usually use a large image patch size to ensure that enough morphological information is included in the patches. In addition, such deep learning methods always require a large training dataset in order to achieve effective classification. Despite the overall good classification results that can be obtained in the whole slide, some regions can still be misclassified, especially those near the tumor-normal margin and those with very few nuclei.^7^

On the other hand, SCC nuclei appear with certain characteristics, such as the variation in nuclei shape, increased nuclei size, atypical mitotic figures, increased number and size of nucleoli, and hyperchromasia.^11^ In addition to the abovementioned morphological features, the color of SCC nuclei, which is related to the optical density of hematoxylin and reflects chromatin condensation, is also an important factor for histological diagnosis.^12^^,^^13^ Nandini and Subramanyam^14^ carried out computer-assisted microscopic image analysis to evaluate the correlation between nuclear features and histologic grading of oral SCC and proved the reliability of nuclear morphometry. In our previous work,^7^ an inception-based convolutional neural network (CNN) was implemented for whole-slide cancer detection in an SCC dataset of 156 patients and a gradient class-activated map (grad-CAM) technique was applied to visualize the critical components in the input images. Our results showed that the network made decisions by looking at the nuclei in the image patches and identifying a few highly suspicious cancerous nuclei. Both studies indicate that the nuclei provide critical information for the classification. Such cancer-related features may improve the diagnosis in suspicious regions. In this study, we hypothesize that by classifying nuclei identified and extracted from the digitized images of histologic slides, we can achieve SCC detection in the histologic slides. Tens of thousands of nuclei with varied shapes, sizes, and colors can be extracted in even one histologic slide and this provides a large amount of training data for a neural network. It can also simplify the network architecture compared with those used for whole-slide image classification with the reduced complexity of anatomical structures in the image patches.

Nuclear segmentation and classification in histologic images have many applications but remain challenging tasks with only the color and shape information. Hyperspectral imaging (HSI) is an optical imaging technology that acquires a three-dimensional (3D) data cube with two spatial dimensions and one spectral dimension. In other words, HSI is able to provide the morphological information and abundant spectral information of nuclei within one image modality. Besides, utilizing HSI in microscopy can extend the three-band RGB color information of the histologic slide into a wide spectral dimension, which possibly offers more details to improve the classification performance. HSI has the potential to serve as a tool to improve the effectiveness and accuracy of pathologic diagnosis. It gained increasing attentions in recent years and was investigated in many studies.^15^^–^^23^ Wang et al.^24^ used hyperspectral images to identify lymphoblastic leukemia cells by applying a marker-based neural network classification along with spectral and spatial feature extractions and obtained an accuracy of 92.9%. In our previous study, Ortega et al.^25^ implemented a CNN for breast cancer cell detection in digitized hyperspectral histological images. Despite the small dataset used in this study, the comparison between HSI and RGB showed the potential of HSI to improve the classification performance. Kopriva et al.^19^ evaluated the feasibility of HSI for the diagnosis of colon cancer metastasis in the liver from five H&E stained specimens collected from the same patient. Spectral angle mapper was used for a pixel-level classification in the hyperspectral histological images and yielded accuracy between 86.85% and 96.92%. Nakaya et al.^18^ conducted colon cancer detection in H&E-stained specimens by classifying the average spectra of nuclei with a support vector machine (SVM). Unsupervised clustering methods were implemented by Khouj et al.^26^ for ductal cancer detection using HSI. The results of the abovementioned works all proved the usefulness of the spectral information from the hyperspectral histologic images. However, the classification in these studies was implemented either by manually annotating and extracting nuclei from the images, which was extremely time-consuming, or based on the spectra from the whole slide. Using the spectra of the entire slide greatly increase the total volume of dataset, especially when the images are acquired with high magnification. Even though SCC nuclei carry many significant cancer-related features^11^ and have been proved to play a critical role in whole-slide cancer detection,^7^ only using the spectra of the SCC nuclei with a simple spectra-based classification would not achieve a very good detection rate for SCC,^27^ mainly due to the spectral disparity of different SCC nuclei with a large shape variation. Moreover, the hyperspectral microscopy systems in many studies were based on a line-scanning hyperspectral camera, which has to be synchronized with a motorized stage.^18^^,^^28^^,^^29^ A spectral-scanning hyperspectral microscopy system, which utilized a monochromator as the spectral-scanning component, was developed for oral cancer diagnosis.^30^ Both of the abovementioned systems needed a tradeoff between system complexity and resolution.

In this study, we investigate the automatic detection of head and neck SCC nuclei in hyperspectral histology images, as well as the feasibility of cancer region identification in histologic slides based on nuclei detection. First, we utilized a custom-made hyperspectral microscopic imaging system, which is able to acquire both HSI and RGB images and does not require any motorized stage or spectral scanning component. The compact design is convenient for clinical use, and the combination of two image modalities is easier for pathologists to accept since RGB histology imaging is the current routine in the workflow. Second, a simple-yet-effective automatic nucleus extraction method based on principal component analysis (PCA) is proposed to locate and extract nuclei from the hyperspectral histological images, with no need for manual pathologist annotation or complicated segmentation network training. Third, we carried out spectra-based classification of the extracted nuclei using SVM, as well as patch-based classification using a CNN, to investigate the ability of HSI to discriminate cancerous nuclei and normal nuclei for SCC. In our previous study,^27^ nuclei classification using HSI patches and HSI-synthesized RGB patches were compared. In this work, both HSI and RGB images were acquired in the same setting. Therefore, we were able to compare the classification performance using HSI and real RGB patches of the nuclei to evaluate the usefulness of the extra spectral information in HSI. We compare the classification performance using the spectral information, spatial information, and both information of nuclei, to analyze how they contribute to the differentiation of nuclei, respectively. To understand the usefulness of the rich spectral features in HSI is a critical step for this technology to be accepted and employed in the digital pathology field. The quantitative comparison results reveal the “value” of both the morphological features and spectral features of SCC nuclei, thus potentially promoting the integration of HSI in the pathology workflow. Furthermore, we proposed a cancer detection workflow comprising automatic nuclei segmentation, nuclei classification, and image-wise cancer region identification, which could potentially work as a diagnostic tool and significantly increase the cancer detection rate in some suspicious regions, where a whole-slide classification network may fail to give an accurate prediction.

## 2 Methods

### 2.1 Histologic Slides from Head and Neck SCC Patients

Twenty-six H&E-stained histologic slides were obtained from larynx, hypopharynx, buccal mucosa, and floor of mouth (FOM) of 20 different head and neck SCC (HPV-negative) patients, as we previously described.^7^^,^^31^ There were three types of slides, namely T, N, and TN. The tissue of each TN slide was resected at the tumor-normal margin, containing both cancerous and normal tissues, while a T slide contains only cancerous tissue, and an N slide is normal tissue. The TN slide was first selected for each patient, but for six patients whose TN slide did not contain enough cancerous or normal tissue, a T slide or N slide would be included to balance the data. All slides were previously digitized with 40× objective magnification and manually annotated by a board-certificated pathologist. In this work, we used the annotated cancerous and normal regions in the digital histology images as ground truth for data selection and classification.

**Fig. 2:**
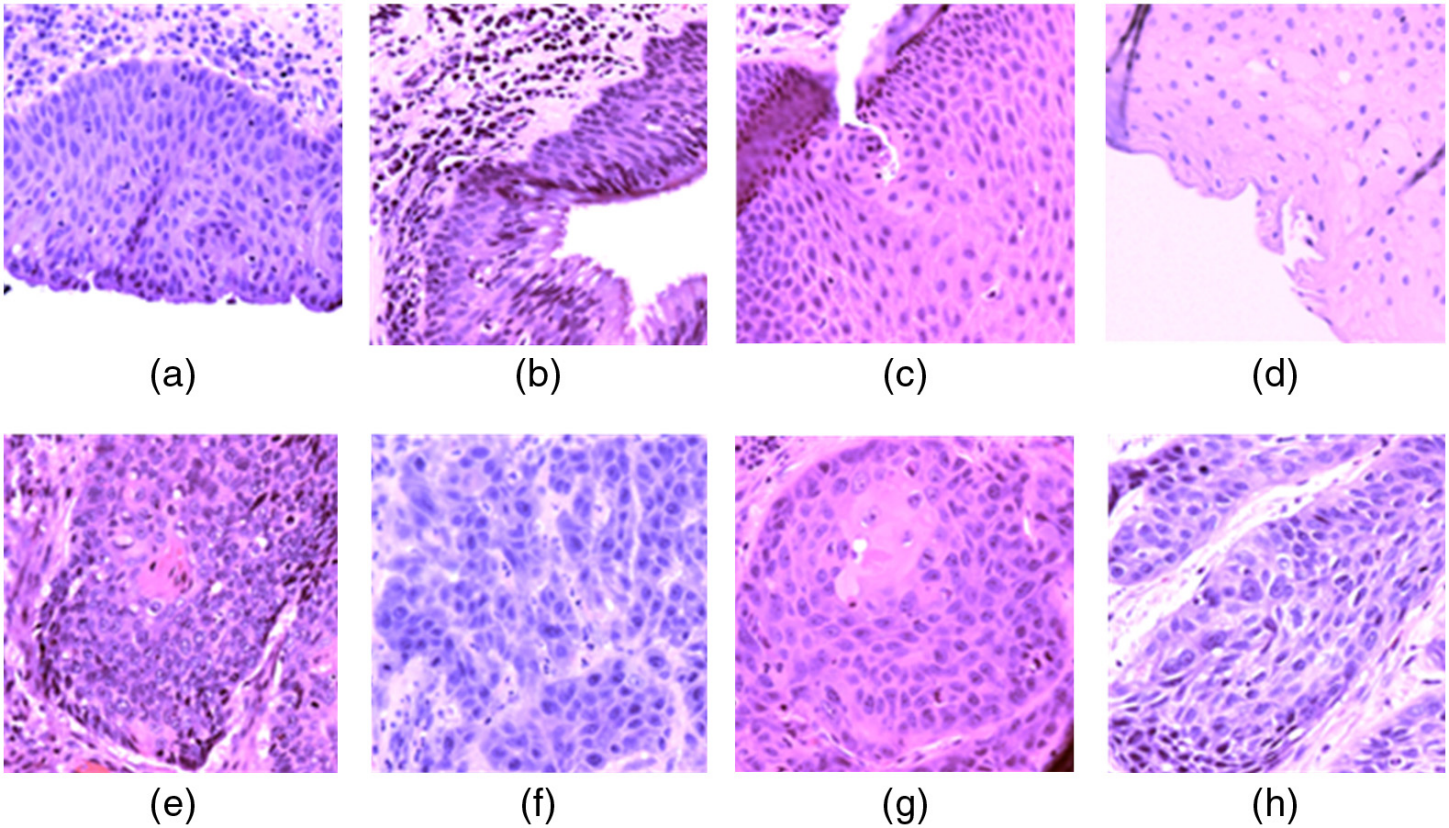
Synthesized RGB images from HSI, which show some representative regions selected for nuclei extraction and quantitative testing. (a)–(d) Normal regions centered at the second and third layers of the healthy stratified squamous epithelium. (e)–(h) Cancerous regions at or close to cancer nests.

In this study, we manually selected regions of interest (ROIs) for both types of tissue for hyperspectral image acquisitions. For each slide, we chose at least three ROIs in the cancerous region annotated by the pathologist who specialized in HNC. We also selected at least three ROIs for the normal tissue. Considering the potential interpathologist variation of the histological diagnosis, both cancerous and normal ROIs were away from the edge of the annotation to avoid any interface area. In addition, there should be adequate nuclei in the images for extraction and classification; therefore, the selected cancerous ROIs were at or close to the cancer nests, where a mass of cells extends to the surrounding area of cancerous growth. The selected normal ROIs were from the healthy stratified squamous epithelium that is far away from the cancerous regions. To make the cancerous nuclei and normal nuclei comparable, we only extracted normal nuclei from the second and third layers of the stratified squamous epithelium, from which the SCC cells originally arise. On average, over 750 nuclei were extracted for each patient, including cancerous and normal nuclei. In total, we collected 257 hyperspectral images (119 normal and 136 cancerous), from which around 15,000 nuclei were later extracted. Figure 2 shows the synthesized RGB images from the hyperspectral images of some representative cancerous and normal ROIs.

### 2.2 Image Acquisition of Histologic Slides

**Fig. 3:**
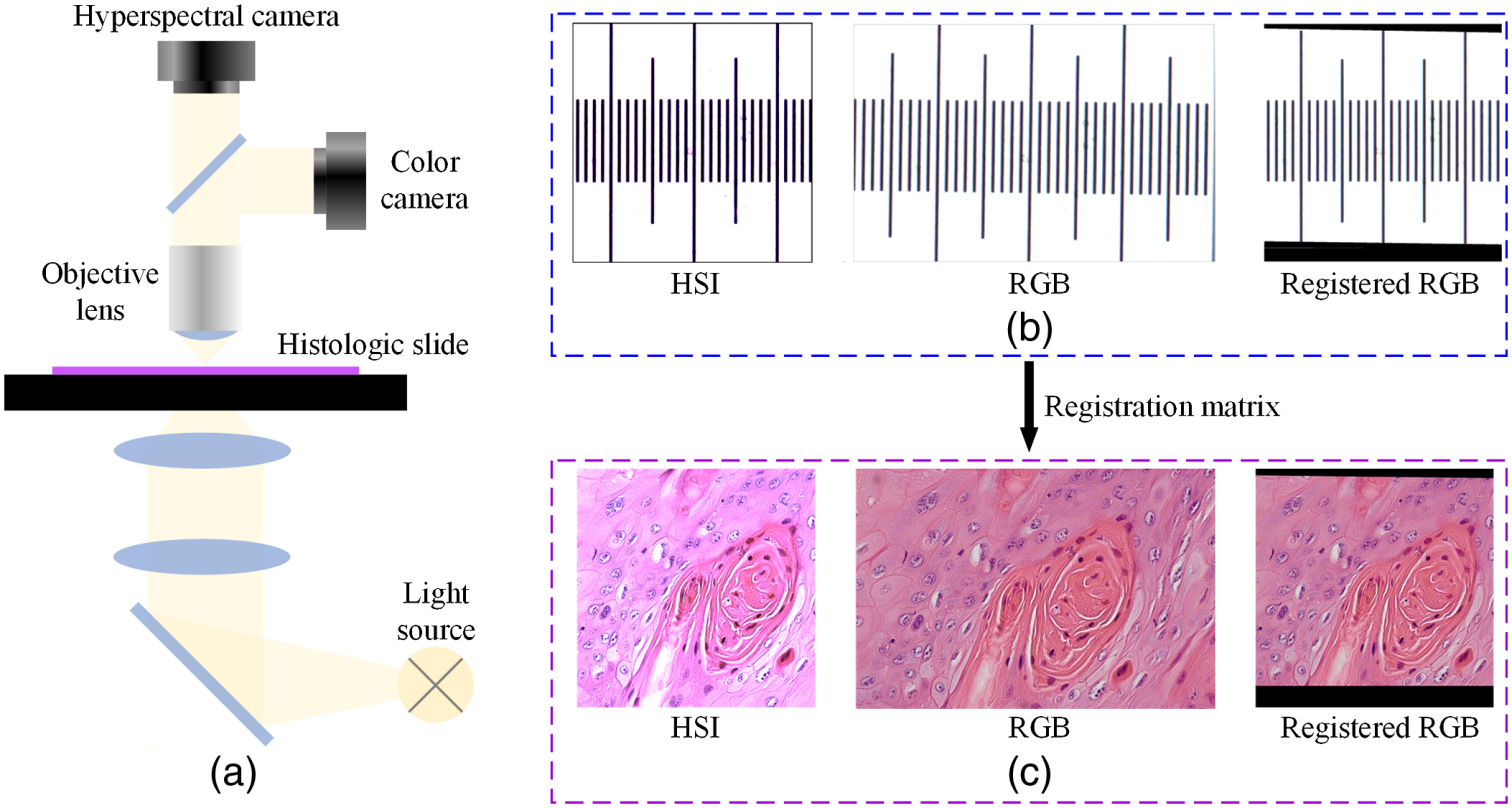
Image acquisition and registration. (a) Diagram of the microscope light path. (b) System calibration by registering the hyperspectral image and the RGB image of the microscopic calibration target. (c) Registering each pair of hyperspectral image and RGB image of the slide by applying the previously obtained registration matrix on the RGB image.

The histological slides were scanned using our custom-made hyperspectral microscopic imaging system, which contains a transmitted-light microscope, a hyperspectral camera, and a color camera, as has been reported in our previous study.^23^^,^^27^ The HSI camera acquired hyperspectral images with 87 bands in the wavelength range from 470 to 720 nm. A color camera was added to the system via an additional optical port on the microscope. The hyperspectral camera and the color camera were tuned to be par-focal, and they shared the same field of view (FOV) with slight differences around the edges due to the different sensor sizes. The light path of the microscope is shown in Fig. 3(a). The spatial size of the hyperspectral images was 2048 ×2048  pixels while that of the RGB images was 3072 ×2048  pixels. The FOV of the hyperspectral camera under a 40× magnification was about 285  μm×285  μm, with a spatial resolution of 139  nm/pixel. Two cameras were synchronized, so that both hyperspectral images and RGB images of the same region were acquired altogether along with the scanning of the slides.

Before acquiring images of the histologic slides, we first implemented system calibration by imaging a microscopic calibration target, as shown in Fig. 3(b). Both the hyperspectral image and RGB image were transferred to grayscale images, and then the RGB-synthesized grayscale image was registered to the HSI-synthesized grayscale image using affine registration with the oriented FAST and rotated BRIEF feature detector^32^ from the OpenCV2 package. The obtained registration matrix was saved as the system calibration matrix, and for each pair of hyperspectral and RGB images that was acquired during slide scanning, we registered the RGB image to the matching hyperspectral image using this matrix, as shown in Fig. 3(c). Note that the “HSI” images in Figs. 3(b)–3(c) are RGB images synthesized from hyperspectral images, which will be explained in the next section.

Before the image acquisition, a blank area on the slide without any tissue or dust was selected. Under the same illumination and focusing setting as during the slide scanning, we first set the white balance of the color camera based on this area, then a hyperspectral image of the same region was taken and used as the white reference image. Dark balance of the color camera was set by blocking the objective lens, and the hyperspectral dark reference image was acquired automatically by the hyperspectral camera along with the acquisition of the tissue images.

Because our hyperspectral microscopic imaging system is based on transmitted-light microscopy, the pixel values in the acquired images are related to the intensity of the transmitted light from the histologic slides. All hyperspectral images of the tissue were calibrated with the white reference image and dark reference image to obtain the normalized transmittance of the slides, as follows:

$$\text{Transmittance}(\lambda )=\frac{I_{\text{Raw}}(\lambda )-I_{\text{Dark}}(\lambda )}{I_{\text{White}}(\lambda )-I_{\text{Dark}}(\lambda )},$$ (1)

where Transmittance(λ) is the normalized transmittance at the wavelength λ, $I_{\text{raw}}(\lambda )$ is the intensity value in the raw hyperspectral image, $I_{\text{white}}(\lambda )$ and $I_{\text{dark}}(\lambda )$ are the intensity values in the white and dark reference images, respectively.

**Fig. 4:**
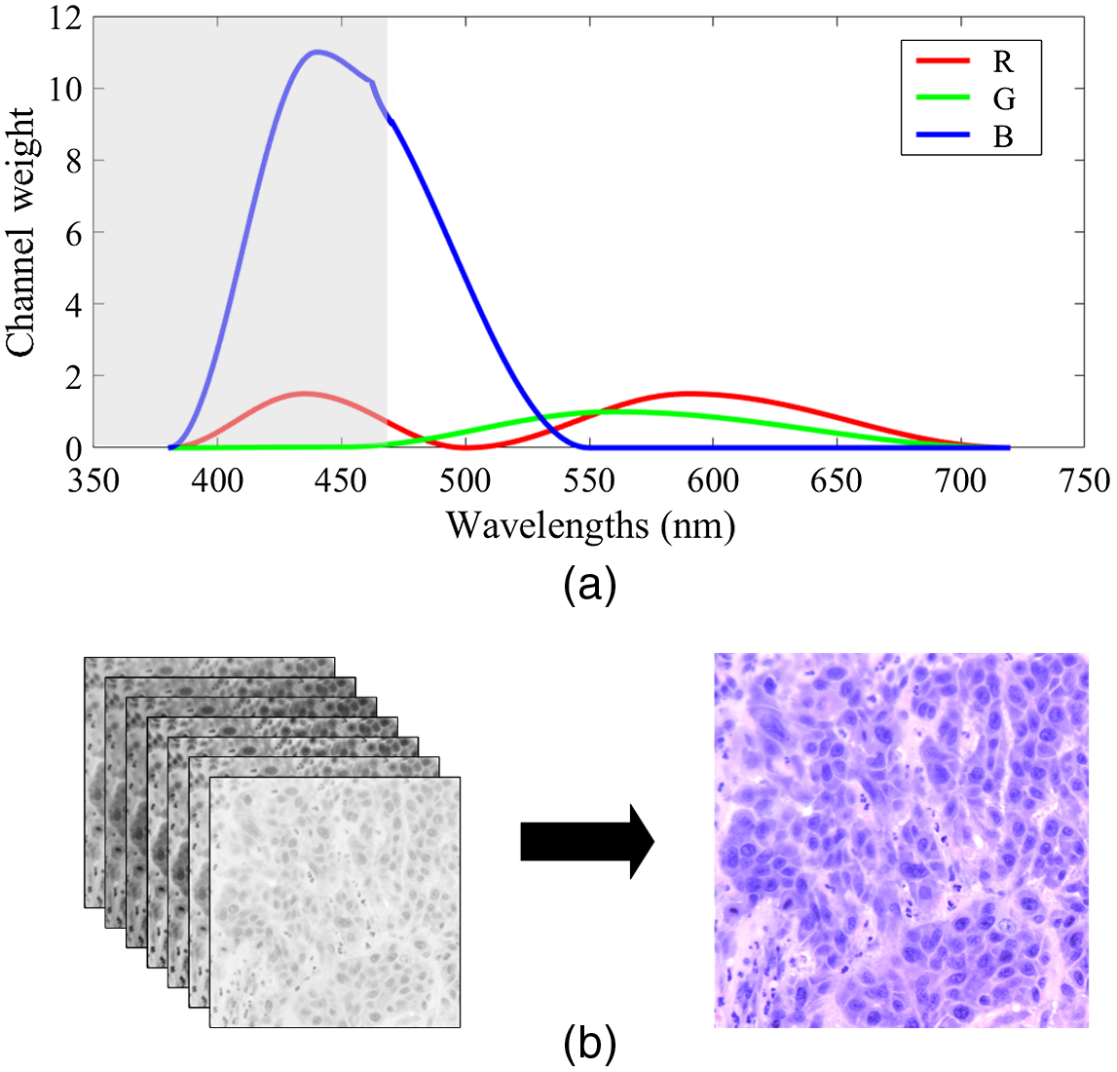
Synthesize RGB image from the hyperspectral data. (a) Customized transformation function for synthesizing an RGB image from a hyperspectral cube, modified from the human eye spectral response for red (R), green (G), and blue (B). The gray area shows the unavailable wavelength range of our hyperspectral camera. (b) A hyperspectral cube and its synthesized RGB image using the transformation in (a).

To visualize the selected ROIs, we synthesized RGB images from the hyperspectral data using our customized transformation function, which was made up of multiple cosine functions, as shown in Fig. 4(a). We developed it based on the spectral response of human eye perception to colors,^33^ but we modified the channel weights for red (R), green (G), and blue (B) due to the absence of the wavelength bands in 380 to 470 nm in our hyperspectral camera. To compensate the absent wavelength bands and balance the colors, we increased the channel weights of red within 380 to 500 nm and the channel weights of blue within 380 to 550 nm. In addition, the colors that we see through our eyes are influenced by the human eye color perception and the spectral signature of the light source. However, the image calibration in Eq. (1) has removed the influence of light source from the hyperspectral images. Therefore, to have the color of the synthesized RGB images as real as possible, we took the light source into consideration and slightly increased the channel weight of the red region from 500 to 720 nm, because the irradiance of the halogen light source is higher in the red-color wavelength range. After applying this customized transformation, the synthesized RGB image was multiplied with a constant of two to adjust the brightness. The synthesized RGB images offer higher contrast and clear visualization of the cellular structures than a single band within the hyperspectral image; and the color was close to the original RGB histologic images, as shown in Figs. 4(b) and 4(c).

### 2.3 Automatic Nuclei Segmentation

SCC nuclei exhibit various cancer-related information. Therefore, by extracting nuclei from the whole slide and implementing cancer detection based on nuclei classification, we can potentially avoid the redundancy of information. Here, we propose a nuclei segmentation method based on PCA. PCA is a multivariate technique for spectral data analysis. It projects the high-dimensional spectral data into a lower-dimensional space and removes the correlation among the wavelength bands. Therefore, it is able to extract the principal component images that have the highest variance, in other words, the highest contrast.^34^^,^^35^ Due to the fact that nuclei are stained by hematoxylin and cytoplasm is stained by eosin,^36^ the different optical absorption properties of the two stains can be used by PCA to form distinctive principal components, where the contrast between nuclei and cytoplasm may be increased.

**Fig. 5:**
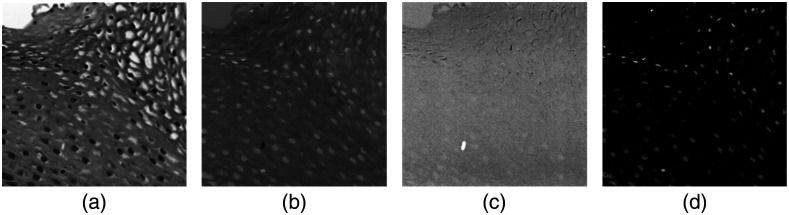
PCA-based nuclei segmentation. (a) The first PC after image normalization (PC1_norm), where pixels of nuclei have lower values than those of cytoplasm and background. (b) The second PC after normalization (PC2_norm), where nuclei pixels have high values than those of cytoplasm and background. (c) The normalized third PC (PC3_norm) that highlights the background. (d) The difference image by subtracting PC1_norm from PC2_norm.

To implementing PCA on the hyperspectral data, each 87-band hyperspectral image was reshaped to a two-dimensional (2D) matrix with 87 vectors. Then, PCA calculation was carried out on the matrix in MATLAB^®^ (MathWorks Inc., Massachusetts, United States). Because of the spectral distinction among the nuclei, cytoplasm, and background, the top three principal components (PCs) highlight these three parts separately, as shown in Figs. 5(a)–5(c). Afterward, we normalized PC1, PC2, and PC3 with their maximum and minimum values

$$\mathrm{PC}i\_\text{Norm}=\frac{\mathrm{PC}i-\min(\mathrm{PC}i)}{\max(\mathrm{PC}i)-\min\mathrm{PC}i)},$$ (2)

where PCi is the i’th principal component, PCi_Norm is the normalized PCi, $\min({\mathrm{PC}}_{i})$ is the lowest value in PCi, and max(PCi) is the highest value in PCi.

Although the nuclei in PC1 seem to be distinct, it is not easy to segment them with a hard threshold. Since the pixels of nuclei in PC1 have a lower value than those of cytoplasm and background, while the pixels of nuclei in PC2 have a higher value, the difference between the normalized PC2 and PC1 (PC2_norm−PC1_norm) yields an image with a high contrast of nuclei and other components, as shown in Fig. 5(d). Generally, the pixels of nuclei have positive values, while those of cytoplasm have negative values, with very a slight difference among various slides. Therefore, a binary mask can be easily generated with the difference image and a hard threshold to segment nuclei from the slides. Considering the general size of nuclei, the segmented components with a very small area were removed. For several overlapped nuclei, we could use a watershed algorithm^37^^,^^38^ to separate them. It is worth noting that the shape of each segmented nucleus in the generated binary mask might not be very precise, but with this method, we were able to easily locate most of the nuclei and extract them from the hyperspectral images.

After applying the PCA-based nuclei segmentation method, the centroids of all segmented nuclei were identified. Because the RGB images were aligned with the hyperspectral images, the identified nucleus centroids also match the locations of nuclei in the registered RGB images. Hyperspectral and RGB patches centered at the centroids were extracted from the images. The patch size was set as 101×101  pixels, which was large enough to include the enlarged SCC nuclei and a few overlapped nuclei that were hard to separate. Afterward, we reviewed all extracted nucleus-centered patches and removed a few outliers that were out of focus. For any nucleus that was extracted from the top or bottom margin area of the registered RGB image, which was filled with black color, the hyperspectral patch and RGB patch of this nucleus would be removed from the dataset. Table 1 shows the final number of cancerous and normal nuclei patches that were extracted from each patient.

**Table 1:** Number of cancerous and normal nuclei extracted from each patient.

Patient #	1	2	3	4	5	6	7	8	9	10
Organ	Larynx									
Normal images	5	6	3	6	6	4	6	4	9	7
Normal nuclei	398	215	105	168	194	277	176	220	444	372
Cancer images	7	6	5	3	3	4	6	8	3	14
Cancer nuclei	551	494	198	158	416	328	279	187	378	779
Patient #	11	12	13	14	15	16	17	18	19	20
Organ	Larynx				Hypo	Buccal mucosa			FOM	
Normal images	11	7	7	10	6	3	5	6	5	3
Normal nuclei	935	318	403	488	312	518	189	405	279	126
Cancer images	6	13	12	7	6	10	5	6	8	6
Cancer nuclei	460	463	695	521	477	465	574	210	302	252

Hypo: hypopharynx; FOM: floor of mouth.

### 2.4 Spectra-Based SVM Classification

**Fig. 6:**
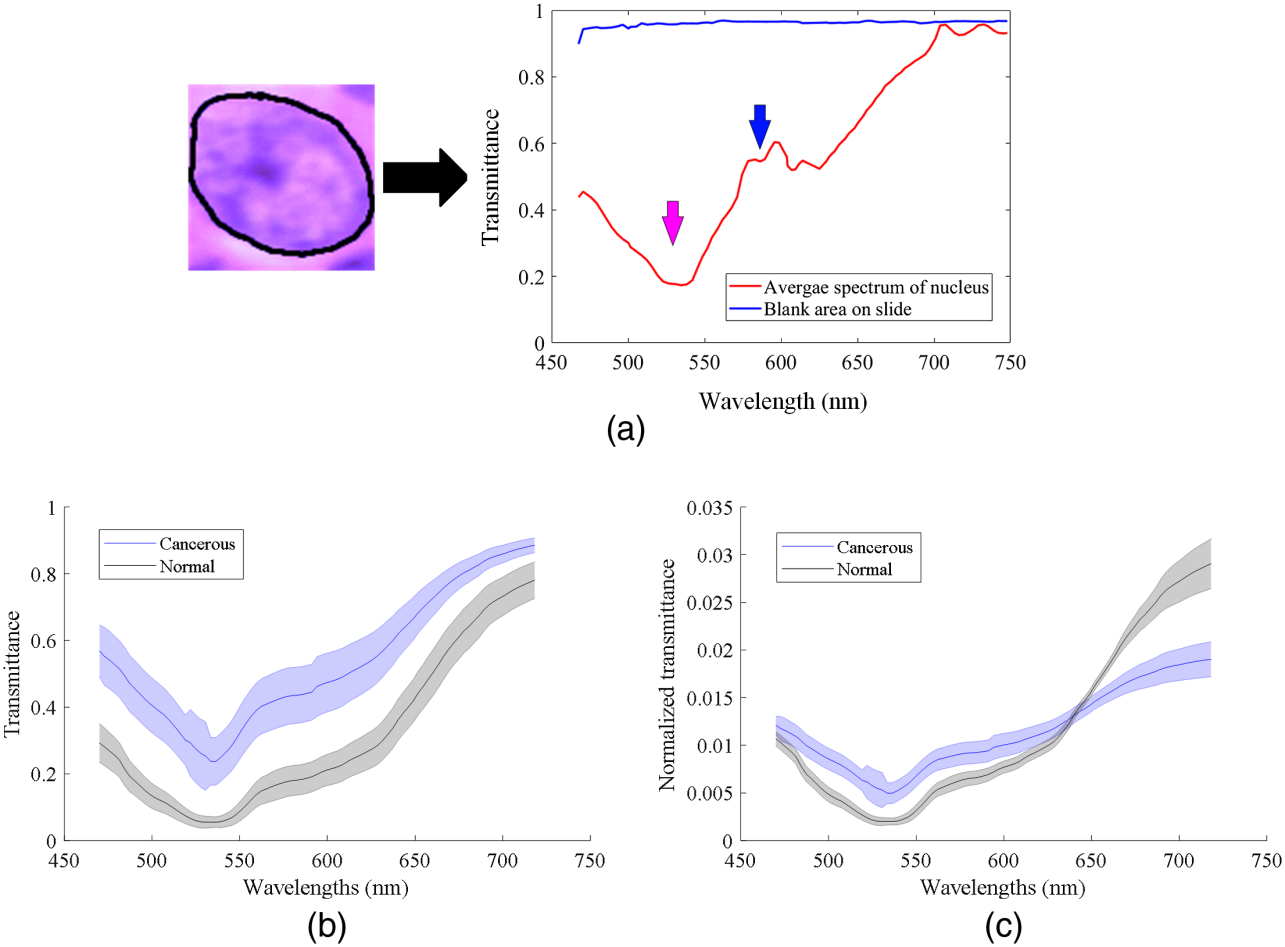
Average nuclei spectra extraction and normalization. (a) The average spectrum of a nucleus in an extracted image patch. (b) Average transmittance spectra of cancerous nuclei and normal nuclei from patient #16. The epithelium in this slide was thick and the nuclei were stained dark, which caused lower values of normal nuclei spectra. (c) Average normalized transmittance spectra of patient #16.

For a straightforward investigation of the distinction ability of nuclei spectra, we obtained the average spectra of all extracted nuclei and carried out spectra-based nuclei classification using an SVM classifier. Although the PCA-based nuclei segmentation method could provide a rough binary mask that helped with the localization of nuclei in the image, the mask was not exactly accurate and might contain some cytoplasm pixels. To have precise spectral signatures of merely the nuclei, we manually and carefully delineated the margin of each nucleus in the extracted nucleus-centered patches, and the average spectrum of all pixels in each outlined nucleus was calculated, as shown in Fig. 6(a). The red curve shows the average transmittance spectrum of the outlined nucleus on the left, and the blue curve is the spectral signature of a blank area on the glass slide with no tissue, which shows an even transmittance across the whole wavelength range after the white reference calibration. The pink arrow indicates the location of the absorbance peak of eosin dye, while the blue arrow points at the absorbance peak of hematoxylin dye.^39^ Because of the thickness variation of the tissue, the amplitudes of nuclei transmittance spectra can be different. Therefore, each average spectrum was then normalized by being divided by a constant, which was the sum of the spectrum at all wavelengths

$$S_{N}(\lambda )=\frac{S(\lambda )}{\underset{\lambda }{\sum }S(\lambda )},$$ (3)

where S(λ) and $S_{N}(\lambda )$ are the transmittance value and normalized transmittance value at wavelength λ.

Figure 6(b) shows the transmittance spectra of cancerous nuclei and normal nuclei from one patient. Specifically, the epithelium tissue in this slide was relatively thick, and the normal nuclei had a very dark color probably caused by overstaining, which resulted in lower values of the transmittance spectra. However, after spectral normalization, the spectral signatures of cancerous nuclei and normal nuclei became more comparable. It could be seen that the values of cancerous nuclei spectra were higher in the wavelength range from 470 to 640 nm, and then became lower after 650 nm. The same trend of nuclei spectra was also observed in many other patients, which means that the spectral signatures of nuclei could provide some distinction.

The normalized average spectra of nuclei were used for the training and validation of an SVM classifier. The SVM was implemented with a radial basis function kernel using MATLAB. Leave-one-patient-out cross-validation was carried out, each time the spectral data from 19 patients were used for training, and the spectra from one patient for validation. Grid search was carried out for SVM hyperparameters C and g over the range of $\log_{2}\;C=\{-1,0,\ldots ,4\}$ and $\log_{2}\;g=\{-1,0,\ldots ,4\}$.

### 2.5 Patch-Based CNN Classification

**Fig. 7:**
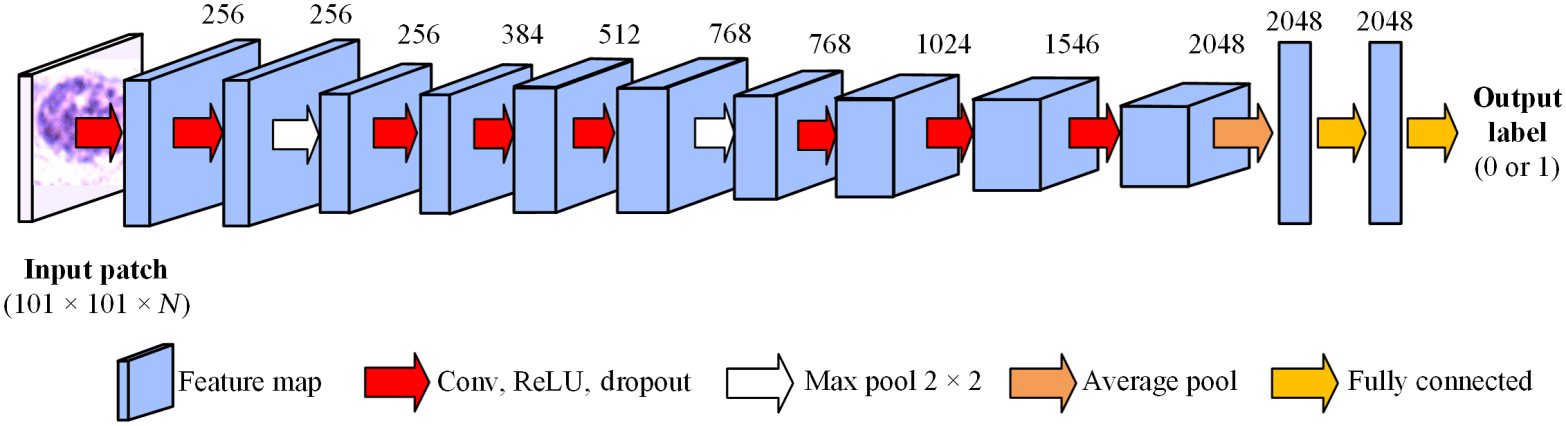
The 2D-CNN for patch-based nuclei classification, with eight standard 2D convolutional layers, two max pooling layers, one average pooling layer, and two fully connected layers. The input size is 101×101×N, where N=3 for RGB patches and N=87 for HSI. Numbers above each figure map show the number of filters.

After the segmentation of nuclei and the generation of nucleus-centered patches, the patches were used for the training, validation, and testing of a 2D CNN. The CNN was implemented using Keras with a Tensorflow backend on a Titan XP NVIDIA GPU. It consisted of eight standard 2D convolutional layers, two max pooling, one average pooling, and two fully connected layers, as shown in Fig. 7 and Table 2. The numbers above the feature maps in Fig. 7 indicate the number of filters. The output had two classes, i.e., cancerous and normal. Except for the first convolutional layer that had a kernel size of 5×5, all other convolutional layers had a kernel size of 3×3. The strides of all convolutional layers were 1×1, and that of the max pooling layers was 2×2. All convolutional layers were initialized with the “Glorot-normal” kernel initializer. The rectified linear unit (ReLU) activation as well as a 10–30% dropout was applied following each convolutional layer. We tried using batch normalization and L2 regularization for the convolutional layers, but they did not improve the training and validation results in this study. The optimizer was Adam^40^ with an initial learning rate of $10^{-5}$ as well as decay parameters beta_1 = 0.9 and beta_2 = 0.999. The loss function was binary cross-entropy. The network was trained with a batch size of 16 for 7 to 27 epochs, depending on when the validation accuracy stopped increasing.

**Table 2:** 2D CNN architecture for patch-based nuclei classification.

Layer	Kernel/strides/padding	Output size
Input	Input image patch	101 × 101 × *N*
Conv	5, 1, “valid”	97 × 97 × 256
Conv	3, 1, “valid”	95 × 95 × 256
Max pool	2, 2, “valid”	47 × 47 × 256
Conv	3, 1, “valid”	45 × 45 × 384
Conv	3, 1, “valid”	43 × 43 × 512
Conv	3, 1, “valid”	41 × 41 × 768
Max pool	2, 2, “valid”	21 × 21 × 512
Conv	3, 1, “valid”	19 × 19 × 768
Conv	3, 1, “valid”	17 × 17 × 1024
Conv	3, 1, “valid”	15 × 15 × 1536
Conv	3, 1, “valid”	13 × 13 × 2048
Average pool	13, None, “valid”	2048
Fully connected (ReLU)	Dense	2048
Fully connected (Sigmoid)	Dense	1

N=87 for HSI patches and N=3 for RGB patches.

As specified in Table 2, the same CNN architecture was used for hyperspectral and RGB patches to compare the classification performance and evaluate the usefulness of extra spectral information in the hyperspectral images. The network that was trained with hyperspectral patches, namely the HSI-CNN, had an input size of 101×101×87, and the one with RGB patches, namely the RGB-CNN, had an input size of 101×101×3. These two networks were tuned separately to avoid bias of optimization, although the learning rate and decay rate turned out to be the same.

All nucleus-centered image patches were split into training, validation, and testing groups to evaluate the network. The same data partition was used for both HSI-CNN and RGB-CNN. We carried out eightfold cross-validation, each time nuclei patches from 14 patients were used for training and those from two patients were used for validation. Four randomly selected patients were left out as an independent testing group, including 2068 nuclei patches extracted from 44 images. The data of one patient have never been used in the training, validation, or testing group at the same time. In addition, all training patches were four times augmented by rotating. Table 3 shows the number of images and nuclei patches that were used in each fold before data augmentation.

**Table 3:** Data partition for cross-validation.

Validation fold	No. of images for training	No. of nuclei for training (before augmentation)	No. of images for validation	No. of nuclei for validation (before augmentation)
1	189	11,003	24	1658
2	193	11,569	20	1092
3	196	11,446	17	1215
4	189	11,779	24	862
5	180	10,688	33	1973
6	176	10,485	37	2176
7	177	10,554	36	2107
8	191	11,283	22	1378

For validation and testing, nucleus-centered patches were also four times augmented by rotating and reflecting. Then, the average probability of each nucleus-centered patch was calculated across all the four augmented versions of the patch. The optimal threshold levels were determined based on the receiver operator characteristic curves for validation data in different folds, and the same threshold was applied to all validation data in the same fold. Finally, the network with the best validation results was selected and tested on the testing data group.

### 2.6 Image-Wise Classification

When doing whole-slide image classification for cancer detection, the appearance of suspicious cancerous nuclei in the image patches is a key factor for the network to make cancer prediction, even if the network was not trained to look for nuclei on purpose.^7^ But with the relatively low magnification (usually 10×), it may miss some of the morphological features and spectral information of nuclei, especially for the regions where few nuclei exist, thus leading to some misclassification. Theoretically, a region should be diagnosed as cancerous even if only one cancerous cell is found in it. Therefore, we propose to use the cancerous nuclei detection results for an image-wise cancerous region identification, which can potentially assist the diagnosis of the slide, especially in the regions where a whole-slide classification network is not able to achieve a precise decision making. Moreover, the FOV under a high magnification is small (<0.3  mm×0.3  mm in this study), so the resolution of an image-wise classification would still meet the need for tumor margin detection.

In this study, image-wise classification was implemented based on the percentage of cancerous nuclei detected in each image. After extracting the nuclei patches using our proposed PCA-based segmentation method and carrying out patch-based nuclei classification using the CNN, the percentage of detected cancerous nuclei among all nuclei within each hyperspectral image was calculated. If the percentage of cancer nuclei in this image was above a certain threshold, the corresponding region on the histological slide would be considered as cancerous. Theoretically, a region should be taken as cancerous even if only one cancer cell exists there. However, considering the possible false positives in nuclei classification, we employed five different thresholds with relatively small values (1%, 5%, 10%, 20%, and 30%), and counted how many images in the validation data groups were correctly classified. The threshold value that resulted in the best image-wise classification among the validation data would finally be applied to the testing data. It is worth noting that in this study, the number of segmented nuclei from different images can be different, e.g., from 6 nuclei per image to near 200 per image, depending on the major histological structure in the image. For example, some images of the cancerous tissue may contain a large SCC pearl, or much more lymphocytes than SCC nuclei due to chronic inflammation, resulting in a very small number of nuclei in the images. For images of the normal tissue, thin epithelium could also result in a small number of extracted nuclei. Therefore, the threshold determined for image-wise classification based on images with different base numbers of nuclei should work for any region, even those that get misclassified due to the small quantity. We counted the number of images in the validation groups with a different number of nuclei detected from them. In total, there were four images with very few (<10) nuclei, 12 images with 11 to 20 nuclei, 90 images with 21 to 50 nuclei, 88 images with a decent number (51 to 100) of nuclei, and 19 images with >100 nuclei.

### 2.7 Evaluation Metrics

Before the image acquisition, we carefully selected imaging ROIs in the histological slides according to the manual reference standard of pathologists specialized in HNC. Cancerous regions were selected from or close to the cancer nests, and normal regions were chosen from stratified squamous epitheliums far from the identified tumor-normal margin. In addition, all segmented nuclei were reviewed to make sure that nuclei extracted from cancerous regions were truly cancerous and those from the normal regions were truly normal. After the nuclei extraction, we looked through the nucleus-centered image patch dataset and removed the outliers.

In this study, we use the area under the receiver operating characteristic curve (AUC) as well as the overall accuracy, specificity, and sensitivity to evaluate the nuclei classification performance, as defined by Eqs. (4)–(6). Accuracy is defined as the ratio of the amount of correctly labeled nuclei to the total number of nuclei in the group. Specificity and sensitivity are calculated from true positive (TP), true negative (TN), false positive (FP), and false negative (FN), where positive corresponds to cancerous and negative to normal

$$\text{Accuracy}=\frac{\mathrm{TP}+\mathrm{TN}}{\mathrm{TP}+\mathrm{FP}+\mathrm{TN}+\mathrm{FN}},$$ (4)

$$\text{Sensitivity}=\frac{\mathrm{TP}}{\mathrm{TP}+\mathrm{FN}},$$ (5)

$$\text{Specificity}=\frac{\mathrm{TN}}{\mathrm{TN}+\mathrm{FP}}.$$ (6)

## 3 Results

### 3.1 Nuclei Segmentation Results

**Fig. 8:**
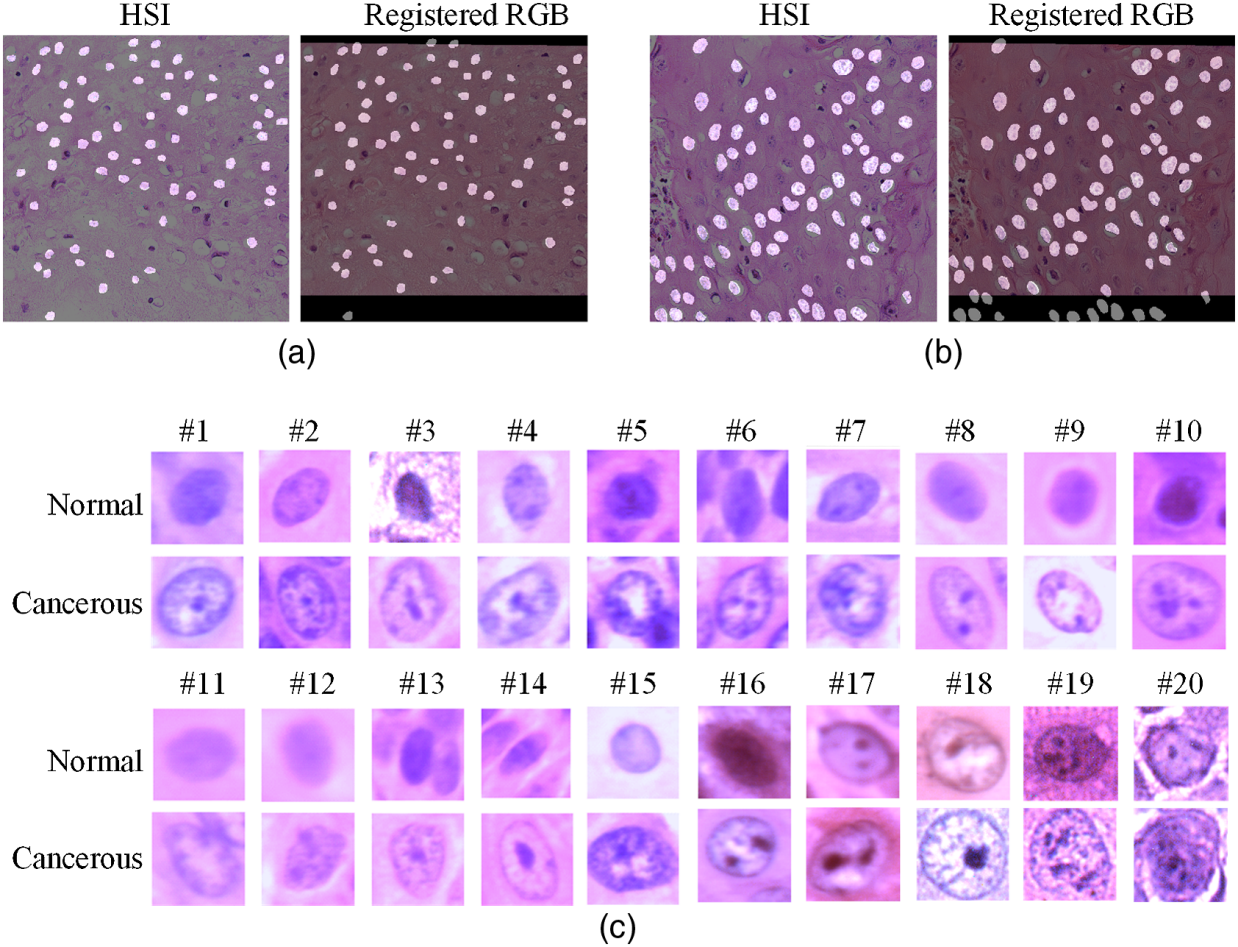
Nuclei segmentation results. (a) and (b) Nuclei segmentation in normal tissue and cancerous tissue, respectively. The segmentation mask obtained using hyperspectral data aligned well with RGB images. Nuclei patches extracted from the top and bottom of registered RGB images were later removed. (c) HSI-synthesized RGB image patches of representative cancerous and normal nuclei were extracted from each patient.

With the proposed PCA-based nuclei segmentation method, binary masks of nuclei were generated, which could separate nuclei from the cytoplasm and background, as shown in Fig. 8. The Fig. 8(a) shows the masks of normal nuclei in the images of healthy epithelium, and Fig. 8(b) shows the masks of cancerous nuclei in cancerous tissues. Because of the slight spectral distinction and the small size, lymphocytes were not segmented. The results show that our proposed method can segment most nuclei in the image. Due to the thickness of the tissue slide, some nuclei at different layers might be out-of-focus and were not as distinct as the in-focus nuclei, and these blurry nuclei were hardly segmented. But this could potentially be solved if automatic focusing and image quality enhancement methods for hyperspectral microscopic imaging can be improved. Figure 8(c) shows representative cancerous and normal nuclei from 20 patients.

### 3.2 Nuclei Classification Results Using Only Spectral Information

The SVM classification using normalized average transmittance spectra of extracted nuclei obtained an average accuracy of 0.68, as well as 0.74 sensitivity and 0.54 specificity. Although the spectra-based classification did not reach a very high accuracy, it did show some distinction ability of the nuclei spectra. Because SCC cells originate from the stratified epithelium, where we obtained our normal nuclei, it is reasonable that the two types of nuclei have a similar shape in their spectral signatures. In addition, the shape variation, size change, and staining variation of SCC nuclei as well as the different cell mitosis stages could all introduce disparities to the spectra among different cancerous nuclei. The spectra-based classification was only carried out for a straightforward investigation of whether the spectral signatures of nuclei would change along with cell carcinogenesis. That being said, there were five patients, i.e., #3, #8, #10, #13, and #18, who got a fairly good accuracy above 0.85. Their spectral signatures of cancerous nuclei and normal nuclei all showed very obvious differences and were in line with the trend as previously shown in Fig. 6(c). In addition, two patients, i.e., #9 and #12, got extremely bad classification results with an accuracy <0.4. In the next section, we compare the patch-based classification results using the RGB patches (spatial and color information) and hyperspectral patches (spatial and spectral information) and see if their spectral difference brought a significant impact on classification.

### 3.3 Nuclei Classification Using Spatial and Spectral Information

**Fig. 9:**
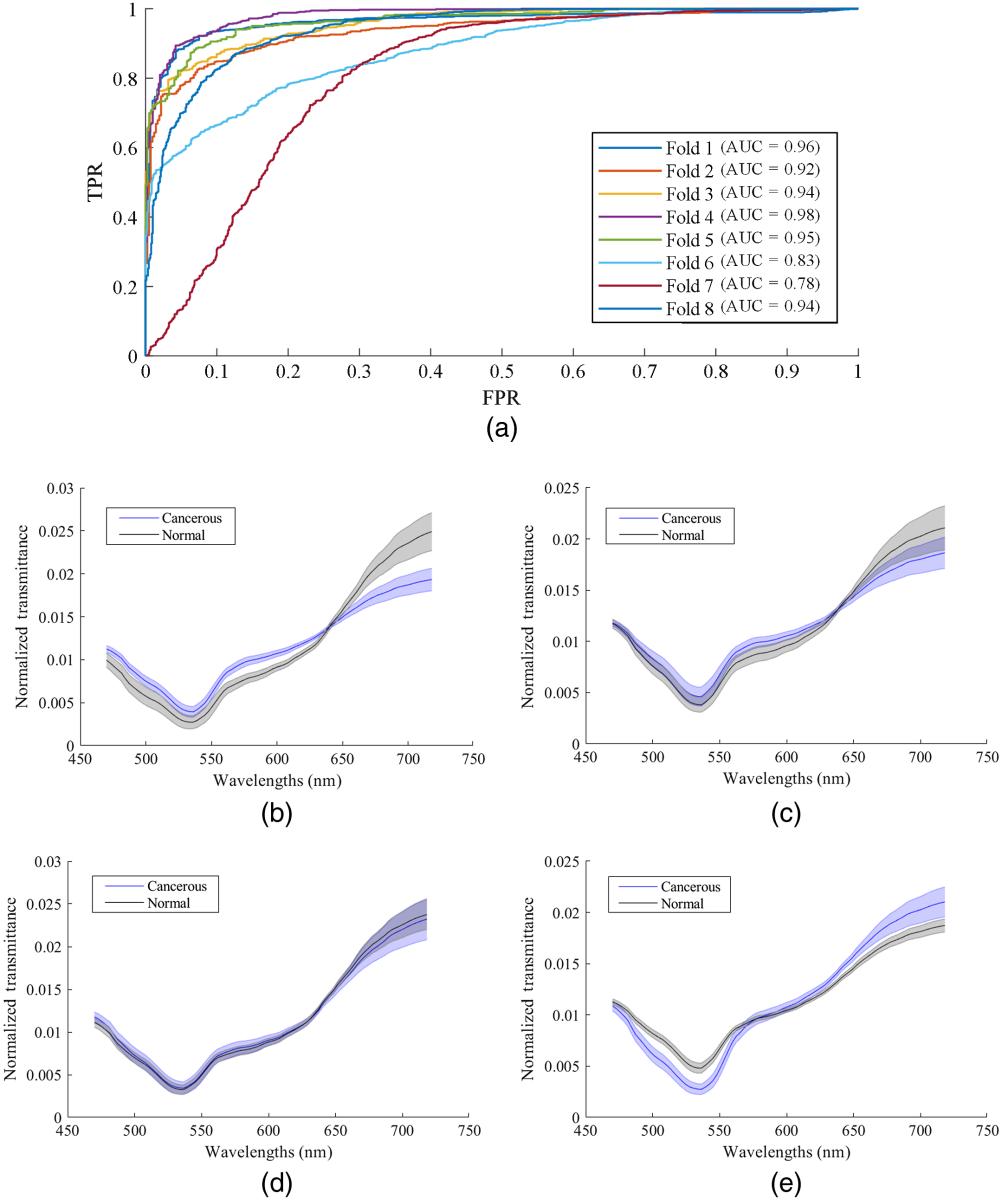
Validation performance and average spectra of cancerous nuclei and normal nuclei from different patients. (a) Receiver operating curves of eight validation folds with corresponding AUC values. (b) Average spectra of patient #13, who had good classification accuracy using the spectra-based SVM (0.91), RGB-CNN (0.97), and HSI-CNN (0.98). The spectral of two types of nuclei are clearly distinctive. (c) Average spectra of patient #8, who got good spectra-based classification accuracy of 0.88, and significant improvement patch-based classification accuracy using HSI-CNN (0.87) compared with RGB-CNN (0.76). (d) Average spectra of patient #6, where spectra-based classification had a close-to-average performance, but the spectral information in hyperspectral patches still helped improving patch-based classification results. (e) Average spectra of patient #12, who got an extremely low spectra-based classification accuracy probably due to the reversed trend of spectral signatures. This is also the only patient who got lower patch-based classification result using HSI.

In this section, we compare the classification performance using hyperspectral patches and RGB patches of the segmented nuclei, as shown in Table 4. For the validation data, the CNN trained with HSI patches could distinguish SCC nuclei from normal epithelium nuclei with an average AUC of 0.93, as well as 0.89 accuracy, 0.88 sensitivity, and 0.89 specificity. The RGB patch-based CNN achieved an average AUC of 0.88 as well as 0.81 accuracy, 0.82 sensitivity, and 0.81 specificity. In half of the validation patients, HSI-CNN outperformed RGB-CNN with an increased accuracy of 3% to 6%, and in five patients (#3, #7, #8, #11, and #14), HSI significantly improved the accuracy by >10%. It is worth noting that all five patients got SVM classification results no worse than the average, especially patients #3 and #8 got 0.88 accuracy. Interestingly, patients #13 and #18 got high classification accuracy using SVM (0.90), RGB-CNN (0.97 and 0.99), and HSI-CNN (0.98 and 0.99), while patient #9 got the worst performance using all three methods (0.35, 0.64, and 0.64). The other patient that had a very low SVM accuracy of 0.38 was #12, who was the only one that got lower accuracy using hyperspectral patches. It can be seen that spatial and spectral information contributes to the differentiation of SCC nuclei and normal nuclei. The morphological features of nuclei seem to play a more dominant role, but spectral signatures do bring a certain impact to the classification performance. Figure 9 shows the average spectra of cancerous and normal nuclei from four different patients, who (1) got very good classification results using SVM, RGB-CNN, and HSI-CNN, (2) got good SVM accuracy and significantly improved classification performance using HSI-CNN compared to RGB-CNN, (3) had less distinctive spectral signatures and an average-level SVM accuracy but still, HSI outperformed RGB, and (4) had abnormal spectral signatures and a very low SVM accuracy, which resulted in worse classification results when using spectral information together with spatial information.

**Table 4:** Patch-based CNN classification results of 20 patients using HSI patches and synthesized RGB patches.

	Patient #	Method	Accuracy	Sensitivity	Specificity
Validation	1	RGB	0.91	0.99	0.81
		HSI	0.96	0.99	0.92
	2	RGB	0.90	0.98	0.73
		HSI	0.93	0.89	0.99
	3	RGB	0.51	0.33	0.84
		HSI	0.90	0.87	0.95
	5	RGB	0.85	0.81	0.93
		HSI	0.88	0.83	0.97
	6	RGB	0.92	0.95	0.89
		HSI	0.95	0.96	0.94
	7	RGB	0.79	1	0.72
		HSI	0.94	0.99	0.92
	8	RGB	0.76	0.71	0.91
		HSI	0.87	0.72	1
	9	RGB	0.64	0.81	0.49
		HSI	0.64	0.56	0.72
	10	RGB	0.86	0.93	0.71
		HSI	0.89	0.87	0.93
	11	RGB	0.60	0.33	0.75
		HSI	0.81	0.86	0.78
	12	RGB	0.85	0.77	0.99
		HSI	0.75	0.89	0.53
	13	RGB	0.97	0.96	0.98
		HSI	0.98	0.96	0.99
	14	RGB	0.75	0.97	0.55
		HSI	0.87	0.97	0.77
	15	RGB	0.79	0.75	0.85
		HSI	0.85	0.81	0.92
	17	RGB	0.89	0.90	0.78
		HSI	0.95	0.95	0.96
	18	RGB	0.99	1	0.98
		HSI	0.99	0.98	1
Testing	4	RGB	0.59	0.91	0.29
		HSI	0.83	0.67	0.95
	16	RGB	0.91	0.82	0.98
		HSI	0.91	0.81	0.99
	19	RGB	0.65	0.99	0.21
		HSI	0.89	0.86	0.93
	20	RGB	0.82	0.75	0.98
		HSI	0.63	0.53	0.83

For the four testing patients, HSI has achieved an average performance of 0.89 AUC, 0.82 accuracy, 0.72 sensitivity, and 0.93 specificity. The CNN trained with RGB patches achieved an average performance of 0.86 AUC, 0.74 accuracy, 0.87 sensitivity, and 0.62 specificity. Although HSI had a lower average sensitivity than RGB, its overall classification performance (accuracy) was significantly better. For patient #16, the spectral signatures of cancerous and normal nuclei were obviously differentiable, and both networks got very good results with a 0.99 AUC. Except patient #16, the classification results for the other three patients using RGB patches and hyperspectral patches were very different. For patients #4 and #19, most SCC nuclei did not show as much size change and shape variation as in other slides, hence the spatial information was insufficient for nuclei differentiation, while using the spectral information in the hyperspectral patches compensated for this insufficiency and significantly improved the results. Therefore, the HSI-CNN achieved an AUC above 0.96 and over 20% improvement in accuracy in both patients. Patient #20, on the contrary, was negatively impacted when using the spectral information, which resulted in very low accuracy of 0.63 as well as an AUC of 0.62. By looking at the average spectra, we found that patients #20 and #12 had the same issue of a reversed spectral trend. The reason that caused this change of spectral signatures was uncertain. One possible explanation is the color variation, which we will in the future improve our network to take care of. However, it is worth investigating whether specific pathological features could possibly result in this change.

### 3.4 Image-Wise Classification Results

In this study, the goal of distinguishing cancerous and normal nuclei is to facilitate automatic cancer detection in the histological slides. Therefore, we carried out image-wise classification based on the percentage of cancerous nuclei detected in the image to locate cancerous tissue. For each single hyperspectral image, the number of nuclei that were classified as cancerous ($N_{C}$) and the total number of segmented nuclei in this image ($N_{\text{Total}}$) were calculated, and the ratio of the two numbers $N_{C}/N_{\text{Total}}$ was used as to identify cancerous regions. Technically, the region should be counted as cancerous if there’s even one cancerous cell in it. However, considering the false positives, we tried five different thresholds for the percentage of cancerous nuclei, namely 1%, 5%, 10%, 20%, and 30%, on the validation data. The number of correctly classified images with each threshold is shown in Table 5. Although using a larger threshold such as 20% could result in more correctly classified images, it also increases the risk of false negatives. It is worth noting that most of the misclassified images were from patients #9 and #12, where the classification performance was generally not very good. As a result, the threshold of 10% was selected and applied to the 44 testing images from four different patients. All 27 cancerous images as well as 15 normal images were correctly identified, resulting in a total accuracy of 0.95. The two normal images that were classified as cancerous were both from patient #20. The percentage of detected cancerous nuclei in these two images was both slightly above 20%. Despite the low sensitivity of patient #20, all cancerous regions were correctly labeled.

**Table 5:** Image-wise classification results in the validation group using different thresholds.

Threshold (%)	Number of correctly classified images		
	Cancerous (total 111)	Normal (total 102)	Both (total 213)
1	111 (100%)	55 (54%)	166 (78%)
5	111 (100%)	79 (77%)	190 (89%)
10	110 (99%)	93 (91%)	203 (95%)
20	109 (98%)	96 (94%)	206 (97%)
30	107 (96%)	97 (95%)	204 (96%)

## 4 Discussions and Conclusions

Machine learning methods, especially deep learning algorithms, have been an emerging technique in recent years to assist automatic pathological diagnosis to improve the diagnostic speed and reduce the interpathologist variation. Most previous studies were carried out using a large RGB image patch size with low magnification to include enough histological features in the patches.^7^^,^^8^^,^^41^^–^^43^ Relatively complex network architectures were usually needed to implement effective classification for those patches.

HSI can capture a subtle spectral difference caused by molecular changes and is able to provide more information than conventional three-band RGB color information of tissue. It is beneficial for many different microscopic applications, including cancer detection in histological slides. However, due to the current absence of a public comprehensive hyperspectral histological image dataset, it might be difficult to fully investigate whole-slide image classification and cancer detection using HSI. Since the appearance of cancerous nuclei is a critical factor for networks to make cancer prediction, and there are many nuclei in slides to provide sufficient training data for a network, we propose to carry out automatic SCC nuclei detection in histological slides using HSI. In this work, we utilized our custom-made hyperspectral microscopic imaging system to image the H&E-stained SCC histological slides for nuclei detection and cancerous region localization in the slides. We used the annotations drawn by the pathologist as the ground truth and carefully selected normal ROIs from the healthy stratified epithelium tissue and cancerous ROIs from or close to the cancer nests. Hyperspectral images and RGB images that share the same FOV were acquired simultaneously, and then RGB images were registered to the corresponding hyperspectral images using affine registration. Synthesized RGB images were also generated using the hyperspectral data and our customized transformation function to provide better visualization of the ROIs. To avoid using the extra spectral information of cytoplasm and other subcellular components, PCA-based nucleus segmentation method was proposed to extract nuclei from the hyperspectral images. Then, two patch-based 2D-CNNs trained with hyperspectral and RGB patches, as well as an SVM trained with average normalized transmittance spectra of the extracted nuclei, were implemented for nuclei classification. The classification results of SVM and RGB-CNN show that both morphological features and spectral information contribute to the differentiation of nuclei. HSI-CNN outperformed RGB-CNN in 18 out of 20 patients and provided significant improvement in classification performance in seven patients (five validation and two testing). This has proved the usefulness of the spectral information in HSI for cancerous nuclei detection in histological slides. Furthermore, we could find an overall trend that the spectra of normal nuclei have smaller values than spectra of cancerous nuclei within the wavelength range from 460 to 600 nm and higher values within the range from 600 to 750 nm. It was also interesting to find that the reversed spectral trend in two patients had a negative impact on their classification results using HSI-CNN. Although it was not clear what caused the abnormal change of spectral signatures, it proves that the spectral information in HSI does play an important role for nuclei differentiation.

Based on the percentage of cancerous nuclei detected in each image, we implemented image-wise classification for cancerous region identification. The proposed methods for SCC nuclei detection as well as image-wise classification could be used to localize cancerous tissue in the slides using a simple CNN architecture. With high magnification, the spatial size of the FOV is smaller than a required tumor margin. Accurate identification of cancerous nuclei, especially when there are very few in the image, could effectively avoid false negative. Our method can be used to assist the diagnosis of some suspicious regions where a whole-slide classification network fails to give very precise predictions, such as the tumor-normal margin. Our lab is now actively developing an automated hyperspectral microscopic imaging system as well as hyperspectral pansharpening algorithms^44^ to achieve fast whole-slide scanning. In the future, we anticipate developing an automated hyperspectral cancer detection method by implementing hyperspectral whole-slide image classification first and applying our nuclei detection method to refine the classification results in suspicious regions and tumor margins.

Nevertheless, our work has certain limitations. Because we manually acquired images, and the dataset was relatively small. Since hyperspectral images intrinsically contain more features than RGB images, it is possible that a larger training dataset is needed for the CNN to fully “understand and play with” those cancer-related features. This might be the reason for the lower average sensitivity of HSI compared to RGB in the testing data group. For the next step, we need to include more data on SCC nuclei from different patients and organs to better investigate the usefulness of the rich spectral information in hyperspectral images. With the automatic whole-slide scanning hyperspectral microscope system that we are developing, we will be able to further investigate the proposed method in a larger region, and hopefully combine it with whole image classification. Another future work for us is to explore which specific wavelength bands or pathological features in hyperspectral images improved the classification results. Finding out the most useful bands might allow the use of cameras with less spectral bands, which will help reduce the image acquisition time and storage space required for hyperspectral image data. Particularly, we want to quantitatively analyze the spectral signatures and pathological features of the SCC nuclei and see if there are potentially any features that lie in hyperspectral images but are not obvious enough to be seen by the human eye. From the experimental results, the nuclei spectra of most well-classified patients had a general trend that the cancerous nuclei spectra have higher values in the short wavelength range and then turn lower after around 600 nm. It was also observed that the reversed spectral trend in patients #12 and #20 did negatively impact the classification results. However, it was not clear what caused the difference in the spectral signatures of these two patients, and it is definitely worth exploring. In addition, cancer development involves many other cellular components and may cause abnormalities of various cells and biomolecules such as lymphocytes and collagen.^45^^,^^46^ We will look into these components and investigate how HSI can facilitate their pathology analysis. Moreover, by combining HSI with other imaging modalities such as autofluorescence^45^ and polarized light imaging,^47^^,^^48^ more features that the human eye cannot directly see in the H&E stained slides, might be revealed.

In conclusion, our hyperspectral microscopic imaging and automatic machine learning method achieve accurate detection of HNC on histologic slides and can provide a promising tool for many pathologic applications.
