# Automatic detection of head and neck squamous cell carcinoma on histologic slides using hyperspectral microscopic imaging (Erratum)

**DOI:** 10.1117/1.JBO.27.5.059802

**Published:** 2022-05-28

**Authors:** Ling Ma, James V. Little, Amy Y. Chen, Larry Myers, Baran D. Sumer, Baowei Fei

**Affiliations:** a University of Texas at Dallas, Department of Bioengineering, Richardson, Texas, United States; b Tianjin University, State Key Laboratory of Precision Measurement Technology and Instruments, Tianjin, China; c Emory University School of Medicine, Department of Pathology and Laboratory Medicine, Atlanta, Georgia, United States; d Emory University School of Medicine, Department of Otolaryngology, Atlanta, Georgia, United States; e The University of Texas Southwestern Medical Center, Department of Otolaryngology, Dallas, Texas, United States; f The University of Texas Southwestern Medical Center, Advanced Imaging Research Center, Dallas, Texas, United States; g The University of Texas Southwestern Medical Center, Department of Radiology, Dallas, Texas, United States

## Abstract

The erratum corrects the name of the first author listed in a reference cited.

This article [*J. Biomed. Opt.* **27**(4), 046501 (2022) doi: https://doi.org/10.1117/1.JBO.27.4.046501 was originally published on 29 April 2022 with an error in the author name listed for Ref. 30, “Hyperspectral microscopy and cluster analysis for oral cancer diagnosis” by A. Jarman et al. The conference proceedings editor K. K. Tsia was mistakenly listed as the first author of that work, due to an undetected error in the EndNote import.

The error was corrected on 18 May 2022.

